# Comparison between the Effect of 810 nm and 940 nm Diode Laser Irradiation on Histopathological Changes in Iatrogenic Oral Ulcers: an Animal Study

**DOI:** 10.30476/DENTJODS.2021.86623.1202

**Published:** 2021-12

**Authors:** Hooman Ebrahimi, Fatemeh Darvish, Mojgan Alaeddini, Shahroo Etemad-Moghadam

**Affiliations:** 1 Dept. of Oral Medicine, Dental Faculty, Islamic Azad University of Medical Sciences,Tehran, Iran; 2 Oral and Maxillofacial Medicine Specialist, Tehran, Iran; 3 Dental Research Center, Dentistry Research Institute, Tehran University of Medical Sciences, Tehran, Iran

**Keywords:** 810 nm Diode Laser, 940 nm Diode Laser, Inflammation, Oral Ulcers

## Abstract

**Statement of the Problem:**

Considering the relatively high prevalence of oral mucosal ulcers, their fast healing is of significance.

**Purpose:**

This study aimed to histopathologically compare the effects of 810 nm and 940 nm diode laser on the healing of iatrogenic oral ulcers in rabbits.

**Materials and Method:**

In this single-blind experimental study, mucosal ulcers measuring 3mm in diameter and 1mm in depth were bilaterally created in the buccal mucosa of 18 rabbits using a biopsy punch. The defects were irradiated with 810 nm diode laser on the right side and 940 nm diode laser on the left side. Biopsy samples of the same depth were obtained from the ulcers on days 3 and 7 followed by histopathological analysis. The intensity of inflammation was determined on hematoxylin-eosin-stained sections using a four-point scale. Data were analyzed employing the Wilcoxon signed rank test.

**Results:**

The degree of inflammation was not significantly different between the 810nm and 940nm diode laser groups on day 3; but on day 7, animals receiving 810 nm experienced a significantly lower degree of inflammation compared to those treated with 940 nm laser (*p*= 0.028).

**Conclusion:**

When comparing 810- and 940-nm diode lasers, 810 nm irradiation significantly decreased the severity of inflammation in oral wounds created on the buccal mucosa of rabbits in a time-dependent manner.

## Introduction

Ulcers are well defined and usually depressed characterized by an epithelial defect covered with a fibrin clot, which often has a yellow-white appearance. Oral mucosal wounds have a high prevalence and their painful nature interferes with normal oral function [ 1 ]. These lesions may develop due to a variety of reasons such as nutritional deficiency of iron and folate, infections, stress, trauma, gastrointestinal problems, deep fungal infection, cutaneous diseases with oral manifestations, medications and allergens [ 2 ]. 

Oral ulcers are associated with tissue inflammation and it causes pain. Thus, any mechanism that can subside wound inflammation will play a role in pain relief of oral ulcers. Conventionally, wound healing in the oral cavity involves several phases including hemostasis, inflammation, proliferation and maturation. In addition to platelets, neutrophils, monocytes, macrophages, lymphocytes, fibroblasts, collagen and a series of mediators and cytokines such as growth factors, including platelet derived growth factor; vascular endothelial growth factor and transforming growth factor-beta play a role in wound healing. In this regard, growth factors stimulate neutrophils and macrophages, have a mitogenic effect on fibroblasts and cause angiogenesis in the process of wound healing and tissue remodeling. Neutrophils and lymphocytes play a primary role in healing of ulcers, and neutrophils are the dominant cells in the first 48 hours following injury. In addition to wound healing, lymphocytes play a role in cellular immunity [ 3 - 5 ]. Oral tissue healing is challenging; thus, various methods are recommended to accelerate this process and inhibit the impeding factors involved in its delay [ 6 ]. 

Any mechanism that can reduce wound inflammation can play a role in pain relief of oral ulcers. Several studies have evaluated the application of lasers in the healing of oral ulcers like aphthous stomatitis, considering their analgesic, anti-inflammatory and wound healing properties as well as the fact that they cause immune system stimulation and affect physiological tissue status [ 7 - 8 ]. Laser therapy has advantages such as cost-effectiveness, faster formation of granulation tissue, improvement in bone repair, wound closure, and faster re-epithelialization [ 9 - 10 ]. 

Low-level laser therapy (LLLT) is an adjunct modality in many dental fields. It has positive effects on oral hard and soft tissues with minimal complications. Moreover, LLLT is used for alleviation of chronic pain and healing of chronic wounds (such as lichen planus) [ 11 ]. It has been documented that LLLT can efficiently decrease inflammation; however, controversy exists regarding the preferred wavelength of laser for this purpose. Thus, the present study aimed to compare the anti-inflammatory effects of 810 nm and 940 nm diode lasers on oral mucosal wounds.

## Materials and Method

The present study was designed based on the Ethical Guidelines for the Care and Use of Animals for Experimental Procedures outlined by the National Institute of Health (NIH). All procedures of present study were approved by the Animal Ethics Committee of Islamic Azad University- Dental Branch Tehran. (IR.IAU. DENTAL. REC. ethical code:1395-96).

**Figure 1:**
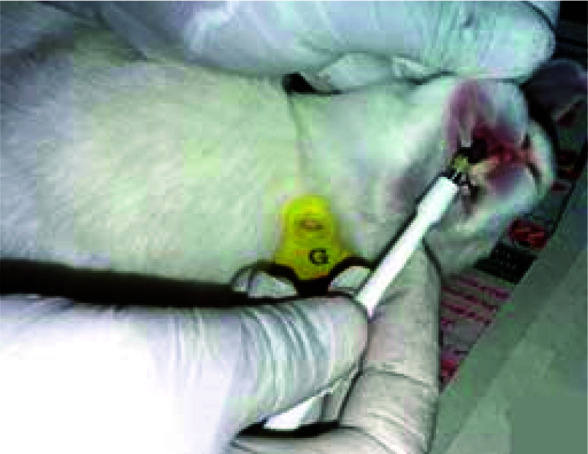
Creating the wound

Eighteen white New Zealand rabbits, weighing 1kg to 2kg, were evaluated in this single-blind experimental study. All animals were kept under 12h light and 12h dark cycle with free access to standard laboratory chow and water at 24°C. General anesthesia was induced using 1.4 mL/kg of 10% ketamine hydrochloride (Alfasan, Holland) and 1 mL/kg of 2% Xylazine HCl (Alfasan, Holland). In addition, 3% mepivacaine without vasoconstrictor was administered for local anesthesia. Mucosal ulcers measuring 3mm in diameter and 1mm in depth were bilaterally created on the buccal mucosa of all rabbits using a biopsy punch (Ind Area, LSC OKHLA, PARA Mont) (Figure 1). 

**Figure 2:**
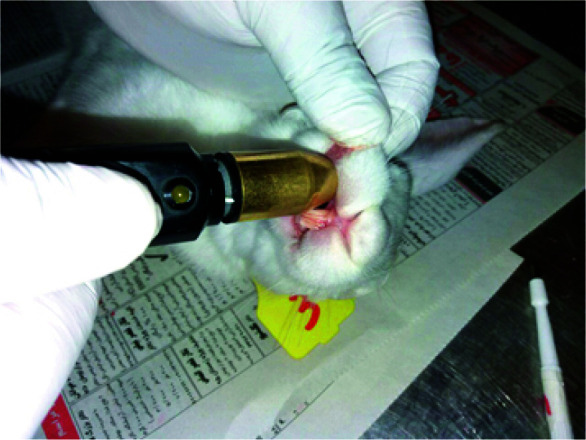
Laser irradiation

**Figure 3:**
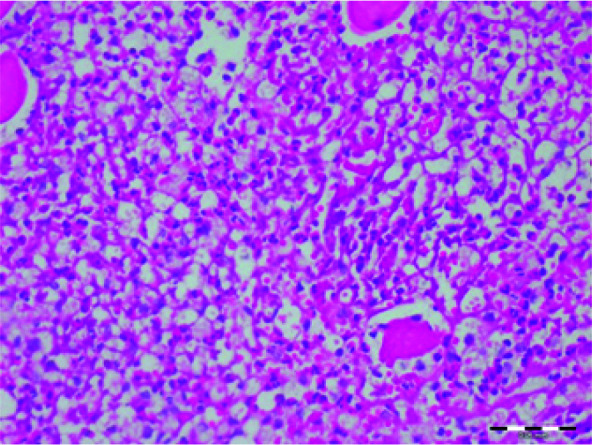
Day 3 sample subjected to 940 nm diode laser (hema-toxylin and eosin stain, original magnification 200×)

**Figure 4:**
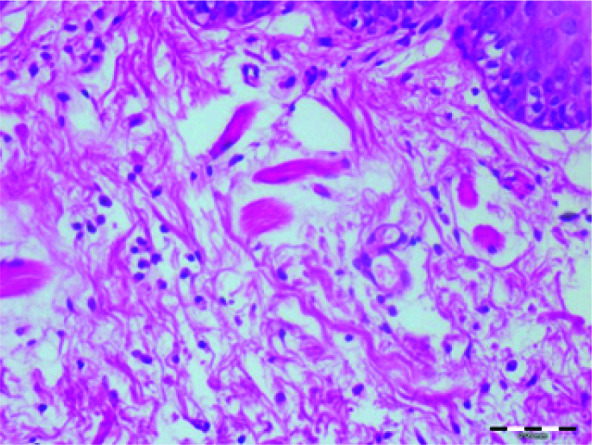
Day 7 sample subjected to 940 nm diode laser (hema-toxylin and eosin stain, original magnification 200×)

The wounds were then subjected to continuous wave LLLT in contact mode (200 mW power) for 10 seconds, with 810 nm diode laser (Thor company, England) used on the left side (group 1) and 940 nm laser (Biolase company, U.S) on the right side (group 2) (Figure 2). Both treatments were used for single session. The probe tip had 1cm diameter and 2.5 J/cm^2^ energy density. Next, biopsy samples were obtained from the wounds of 9 rabbits after 3 days using the same punch. All samples were immersed in 10% formalin and sent to the pathology laboratory for histopathological processing followed by hematoxylin and eosin staining (Figure 3). The same was done for the remaining rabbits after 7 days (Figure 4) [ 12 ]. 

### Histopathological analysis

For histopathological analysis, hematoxylin and eosin- stained tissue samples were microscopically examined by two oral and maxillofacial pathologists under a double-headed microscope (BX51; Olympus, Tokyo, Japan) and any possible disagreements were resolved by consensus. The intensity of inflammation was determined according to Alipanah *et al*. [ 12 ] and reported based on a four-point scale. Scores 1 through 4 indicated the presence of inflammatory cells by 0-25%, 26%-50%, 51%-75% and 76%-100%, respectively [ 12 ].

### Statistical analysis

The degree of inflammation on the right and left sides was compared using the Wilcoxon signed rank test.

## Results

Mean data in the 810 nm and 940 nm laser groups at day 3 and day 7, were separately calculated and categorized to determine the degree of inflammation. The results are presented in Tables 1 and 2.

**Table 1:** Degree of inflammation in the two laser irradiation groups at 3 days

Degree of inflammation	1	2	3	4	*p* Value
Wavelength	0-25%	26-50%	51-75%	76-100%	
810 nm	0	3	0	6	0.441
940 nm	1	1	4	3	

**Table 2:** Degree of inflammation in the two laser irradiation groups at 7 days

Degree of inflammation	1	2	3	4	*p* Value
Wavelength	0-25%	26-50%	51-75%	76-100%	
810 nm	2	5	2		0.028
940 nm	0	4	4	1	0.028

After assessment of inflammation cells, the maximum and minimum average percent inflammation cells in day 3, for laser 940nm was 98.9% and 23.5% and for laser 810nm was 100% and 37.2% and also the maximum and minimum average percent of inflammation cells in day 7, for laser 940nm was 92% and 28.7% and for laser 810nm was 52.8% and 29%.

The degree of inflammation was not significantly different between the laser groups on day 3 (*p*= 0.441), but it was significantly lower in animals receiving 810 n m compared to those irradiated with 940 nm laser (*p*= 0.028).

## Discussion

The effect of LLLT on wound healing is mediated through modification of biological mechanisms, including the induction and synthesis of various growth factors, interleukins and cytokines. Many of the molecules released after LLLT irradiation have a promoting impact on the healing process. For example, increase in basic fibroblast growth factor contributes to the proliferation and differentiation of fibroblasts which are one of the key elements in healing and repair.

These cells synthesize collagen following activation by transforming growth factor beta, which is also increased by LLLT. Another element upregulation via irradiation is the vascular endothelial growth factor with stimulatory effects on neovascularization [ 13 - 14 ]. The application of LLLT for management of mucosal wounds has been previously investigated with results supporting anti-inflammatory, analgesic and bio-stimulatory impacts on oral mucosal wounds [ 15 - 16 ]; however, the optimum irradiation specifications has not been established. The present study demonstrated decreased inflammatory cell count at the site of ulcers, following LLLT application. Although there are some studies that did not compare different laser types, our study exclusively showed the effect of diode laser wave lengths on inflammatory cell reduction. Modulation of cellular inflammation has been corroborated in a systematic review suggesting various processes such as gene expression modification, growth factors, bone-remodeling markers, and inflammatory mediators to be responsible in one way or the other [ 17 ].

Irradiation of surgical extraction sites with 4 cycles daily of 660 nm laser (5 J/cm^2^) in a previous investigation has shown alleviation of pain and inflammation, demonstrating analgesic and anti-inflammatory effects of this intervention [ 18 ]. Their postoperative application of LLLT and resolution of inflammation was comparable to our study. Moreover, similar to the present research, another investigation using the same GA-Al-AS laser with multiples cycles, found consistent reduction in inflammation. Pol *et al*. [ 19 ] evaluated the anti-inflammatory and analgesic effects of LLLT following bilateral extraction of impacted third molars. However, our results are supported by histological analysis, while they used a visual analog scale, to assess inflammation [ 19 ]. 

Other investigations on diabetic ulcers and traumatic wounds have also confirmed the positive effects of LLLT on inflammation and suggested this treatment modality to be easier, more cost effective and with fewer complications. The distinguishing feature of our study was the use of histopathology in the evaluation of inflammation [ 20 ]. Another study conducted on the effect of LLLT on healing of diabetic foot ulcers concluded that LLLT decreases pain and treatment costs in diabetic patients. Their results, similar to others, confirmed the positive effect of LLLT on wound inflammation [ 21 ]. The other study that evaluated the efficacy of 940 nm LLLT and different energy densities on bone healing in an animal model and found no significant difference in the number of bone cells and fibroblasts in different groups at different. This finding revealed that LLLT with different energy densities does not have variable effects on bone healing [ 22 ]. Their study confirmed the effect of LLLT on inflammatory cells but found no significant difference in this regard among different laser energy densities. Their finding regarding the effect of LLLT on inflammatory cells was in agreement with our results. 

According to the results obtained in the present study, a significant difference in the degree of inflammation was observed between the two laser groups on day 7, but not on day 3. We obtained lower inflammatory cell count in animals receiving 810 nm compared to those irradiated with 940 nm. This was in contrast to another study who found a significant continuous reduction in inflammation during days 3, 7 and 14 using 658nm [ 12 ]. This finding appears to be due to the different biological behavior of various wavelengths of laser and their subsequently different effects on inflammation. 

A systematic review on the effects of LLLT on human periodontal ligament fibroblasts concluded that LLLT has positive effects on proliferation of fibroblasts and different types of osteogenic cells and also modulates cellular inflammation [ 17 ]. 

Studies on periodontitis and recurrent aphthous ulcers using 940 nm similar to our setting, also demonstrated reduced inflammation and even reported that one-time irradiation of this wavelength can have immediate lasting analgesic effect [ 23 ]. 

In the current study, two different wavelengths of diode laser were evaluated. Since the biological behavior of LLLT and light changes with different wavelengths, it is expected that the number of inflammatory cells may also change following alterations in this factor. Although both 810 and 940 nm lasers were low-level and have similar behavior with respect to inflammatory cells and collagen synthesis, they demonstrate different quantitative effects on inflammatory cells which can be explained according to the Planck's constant (h). Considering that $E=\frac{\mathrm{hc}}{\lambda }$ , where h indicates the Planck's constant, c shows the speed of light in vacuum and λ exhibits wavelength, the energy of each photon would be a function of its wavelength. Therefore, by changing the wavelength, the biological entity of the photon and subsequently its biomodulation effects would also be altered. 

## Conclusion

Selection of an appropriate laser wavelength is important in LLLT treatment of oral ulcers, since it can affect healing at the cellular level. In comparison to 940 nm, 810 nm diode laser had a greater effect on reducing inflammation of rabbit oral mucosal wounds after one week. There was no significant difference between the two wavelengths on day 3 of treatment. 

## Conflict of Interest

None declared.
